# Energy and Distance Based Multi-Objective Red Fox Optimization Algorithm in Wireless Sensor Network

**DOI:** 10.3390/s22103761

**Published:** 2022-05-15

**Authors:** Rajathi Natarajan, Geetha Megharaj, Adam Marchewka, Parameshachari Bidare Divakarachari, Manoj Raghubir Hans

**Affiliations:** 1 Department of Information Technology, Kumaraguru College of Technology, Coimbatore 641049, India; rajathi.n.it@kct.ac.in; 2 Department of Artificial Intelligence and Machine Learning, Sri Krishna Institute of Technology, Bangalore 560090, India; geethacse@skit.org.in; 3 Faculty of Telecommunications, Computer Science and Electrical Engineering, Bydgoszcz University of Science and Technology, 85-796 Bydgoszcz, Poland; 4 Department of Telecommunication Engineering, GSSS Institute of Engineering and Technology for Women, Mysuru 570016, India; paramesh@gsss.edu.in; 5 Department of ECE, School of Engineering, MITADT University, Pune 412201, India; manoj.hans@mituniversity.edu.in

**Keywords:** cluster head, wireless sensor networks, multi-objective red fox optimization, energy consumption, network lifetime

## Abstract

In modern trends, wireless sensor networks (WSNs) are interesting, and distributed in the environment to evaluate received data. The sensor nodes have a higher capacity to sense and transmit the information. A WSN contains low-cost, low-power, multi-function sensor nodes, with limited computational capabilities, used for observing environmental constraints. In previous research, many energy-efficient routing methods were suggested to improve the time of the network by minimizing energy consumption; sometimes, the sensor nodes run out of power quickly. The majority of recent articles present various methods aimed at reducing energy usage in sensor networks. In this paper, an energy-efficient clustering/routing technique, called the energy and distance based multi-objective red fox optimization algorithm (ED-MORFO), was proposed to reduce energy consumption. In each communication round of transmission, this technique selects the cluster head (CH) with the most residual energy, and finds the optimal routing to the base station. The simulation clearly shows that the proposed ED-MORFO achieves better performance in terms of energy consumption (0.46 J), packet delivery ratio (99.4%), packet loss rate (0.6%), end-to-end delay (11 s), routing overhead (0.11), throughput (0.99 Mbps), and network lifetime (3719 s), when compared with existing MCH-EOR and RDSAOA-EECP methods.

## 1. Introduction

Generally, a WSN is made with a massive amount of sensor nodes that are randomly placed in a coverage region [1]. These nodes take local physical data, process them, and transfer them to a sink or base station (BS) [2]. The BS is connected to the internet in order to raise public awareness of the phenomena. Another key feature of a WSN is the capacity of the nodes to collaborate [3]. Instead of sending raw data to the data fusion node, sensor nodes use their processing capabilities to perform computations and fusion operations locally, transmitting just the data that are needed [4,5]. Wireless sensors with these features are employed in a variety of applications, including surveillance and monitoring [6]. WSNs are used in several applications such as weather forecasting, the defense domain, and various commercial and industrial applications. WSN technology, when compared to existing environmental monitoring techniques, is a green energy-based technology that is more favorable for future applications in effectively detecting environmental fluctuation [7]. WSNs used for environmental monitoring are made up of excess battery-operated nodes that are closely distributed in inaccessible or remote locations [8]. The fundamental problem in WSNs, however, is the sensor nodes’ limited power resources. As, in many cases, the nodes are deployed in hostile environments, it is not possible to recharge or replace their batteries after they have completely depleted their energy [9,10].

As a result, while traditional networks strive for excellent quality of service (QoS), sensor network protocols must prioritize energy conservation, in order to extend the network’s lifetime [11,12]. This topic developed a number of research concerns. The most promising challenge among them is the development of energy-efficient clustering and routing algorithms [13]. Clustering in WSNs entails efficiently grouping sensor nodes into different clusters, with each cluster commanded by a cluster head (CH) [14]. The CHs gather data from all of their corresponding members, combine them, and send them to the BS. Each sensor node belongs to a single cluster and only connects with that cluster’s CH [15]. As a result, effective CH selection is required to balance the CHs’ energy consumption; otherwise, they expire quickly, because of supplementary data aggregation and transmission [16]. Most cluster-based routing algorithms choose CHs at random, or based on prospects, before forming clusters [17]. However, in such instances, all CHs may be concentrated in a small area, a fact that leads to the isolation of certain conventional nodes, potentially causing network failure [18,19]. Moreover, the energy efficiency and quality of service are deliberated as a multi-objective optimization issue, in order to maintain the tradeoffs between network coverage, energy efficiency, and network lifetime; these are analyzed in previous research works. This research work proposes an energy and distance based multi-objective red fox optimization algorithm (ED-MORFO) for overcoming the problems caused by network failure. The research modeled a global search by simulating a red fox searching for prey over land. The local search simulates disguising the prey when hunting for the maxima or minima test functions, which is efficient for overcoming the optimization problems. The algorithm locates the individuals in the direct surroundings of the optimum after a short interval of time, and provides a good advantage for improving the final precision results. The fitness values, such as residual energy, network coverage, distance, and degree of nodes, are considered as the fitness functions for finding the optimal solutions for the selection of nodes. The study’s major contributions are as follows:

- The ED-MORFO-based clustering & routing process was exploited to achieve an energy-efficient procedure, with a variety of node counts for improving the lifetime of the network by minimizing the energy consumption.
- To improve the data transfer dependability, ED-MORFO was employed to reduce energy consumption and improve link superiority across the various sensor nodes.

The structure of this research paper is as follows: Section 2 offers a survey of contemporary strategies in WSN clustering and routing that are energy efficient. Section 3 contains the research’s problem statement. Section 4 explains the preliminaries and the system model. Section 5 contains a block diagram and a description of the suggested approach. Section 6 declares the simulation findings, as well as a comparative study of the proposed method. Finally, in Section 7, the conclusion is expressed.

## 2. Related Work

There are many existing techniques connected to energy-efficient clustering and routing in WSNs that were developed for various applications. A brief evaluation of some contributions to the existing literatures is given as follows:

Seedha Devi et al. [20] propose a cluster-based data aggregation scheme (CDAS) for packet loss and latency reduction in a WSN. The proposed structure has two stages: the aggregation tree structure and the slot planning procedure. In the first step, each CH uses compressive accumulation to collect data from the participants. Previously, the spanning tree process was used to generate the accumulating tree through the sink. In the second stage, latency and packet loss are reserved for analysis, while the acquired data are used to highlight and assign intervals to nodes.

Pattnaik and Sahu [21] present a fuzzy-based clustering approach, as well as an elephant herding optimization (EHO)-greedy method for routing. To save energy, EHO-greedy considers both the permanent and portable sinks. A stable node is randomly positioned diagonally throughout the arrangement, while a portable node shifts into various spots for data collection. A good group of CHs drastically reduce energy use, while also extending lifespan. On the other hand, in some other applications, the addition of more energy-efficient techniques leads to larger WSN zones.

Mahdi et al. [22] demonstrate a packet computation that uses the gray wolf optimizer to select CHs (GWO). The configurations are evaluated based on the expected energy use and the current lingering energy of each hub when choosing CHs. The suggested methodology employs similar clustering in different sequential rounds to improve energy efficiency. In cases where the present cluster process is appropriate, the set-up procedure anticipates loss due to excessive execution of the cluster organization step. The set-up protocol does not take into account the QoS metric when it is separated from the lifetime. There is a variation in an internal component in some of the applications, which causes routing protocol issues.

Zhang and Yan [23] propose the centralized energy-efficient clustering routing protocol for versatile hubs protocol (CEECR), which is designed to reduce energy consumption. It is also used to create optimal clusters by combining node portability and energy efficiency. The CEECR protocol employs a focal control calculation, which is used to create a superior cluster heads (CHs) arrangement with more energy, rather than portability. Furthermore, the best CH for a withdrawn hub is decided based on the aggregated loads. As a result, the CEECR is more energy efficient than its competitors. Higher detached nodes (DNs) emerge as a result of the increased number of mobile nodes (MNs), which results in more data packet loss, and high energy consumption.. Deepak Mehta and Sharad Saxena [24] demonstrate multi-objective, CH-based, energy-aware optimized routing (MCH-EOR) in a WSN, in order to extend its lifetime. The cluster head is chosen based on a variety of criteria, with the primary goal of reducing the number of dead sensor nodes, while lowering energy usage. Following the selection of cluster heads, the sailfish optimizer algorithm is used to choose the best path for transmitting data to BS, increasing the energy efficiency of wireless sensor networks. However, data loss is high and transmission coverage and connectivity factors are not taken into account. G. Rajeswarappa and S. Vasundra [25] developed the red deer and simulation annealing optimization algorithm-based energy-efficient clustering protocol (RDSAOA-EECP) for enhancing the network’s stability and lifetime. This methodology is designed utilizing SA to avoid the battling and roaring stages of RD throughout the escalation development. Furthermore, this integrated optimization receives the possibilities of the suggested features to produce the ideal CH, as well as the ideal BS position, in order to improve energy efficiency. However, there is still opportunity for advancement in terms of exploratory and exploitative skills to extend the lifetime of a network.

## 3. Problem Statement

- In energy-efficient routing, the key problem is losing connectivity during data transmission while utilizing energy control mechanisms [21].
- Data loss occurs as a result of a defective network. As a result, conflicts arise in effective data transmission from the clustering nodes [24].
- Due to insufficient power being given to the nodes, the advancing of numerous subjects and node energy consumption are important concerns in WSNs, as they show higher redundancy and energy consumption [23].

### Solution

A metaheuristic or classical technique is extremely desirable for obtaining a more efficient solution to the clustering and routing problem with the above-mentioned difficulties. As a result, this research develops ED-MORFO-based clustering and routing algorithms for WSNs that reduces the sensor nodes’ energy consumption, in order to extend the network’s life time.

## 4. Preliminaries

The existing methods stated in the related works are not capable of offering an improved solution because of the deficiency in the consideration of essential parameters during fitness function derivation. The proposed ED-MORFO employed suitable consideration of crucial constraints such as residual energy, distance, network coverage, node degree, queue length, and link quality. Due to the various fitness function parameters, the energy-efficient results are accomplished in proposed ED-MORFO when compared to existing methods. The major goal of this study was to develop an energy-efficient routing system for sending the data packets. This section explains the network model, energy model, and an overview of RFO used in this cluster-based routing.

### 4.1. Network Model

**Figure 1:**
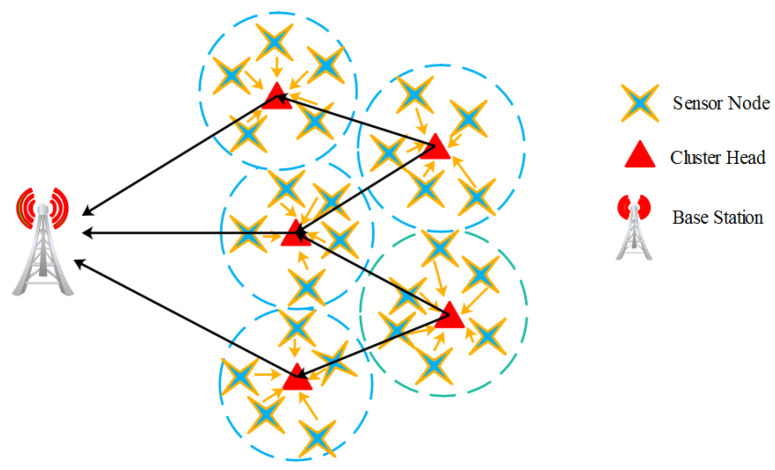
Organization of cluster-based WSN.

The cluster-based WSN [26] was organized, as shown in Figure 1. The following assumptions were utilized to develop the network model:

- In terms of processing time and energy, WSN sensors are identical to one another;
- The Euclidean distance principle is deliberated to calculate the distance between the sensors;
- Once the distance is calculated, the sensors are located in the network region;
- BS assumed the residual energy and distance of the nodes to pick the CHs, using an appropriate CH selection technique. Furthermore, a routing procedure is used to determine the communication route from the CH to the BS.

For the WSN setup, all of the above qualities and constraints were taken into account. By comparing the received signal strength, the distance between the BS and other nodes was calculated. As a result, with location services such as GPS, no additional system was required. Similarly, a node joined the cluster whose CH it was closest to.

### 4.2. Energy Model

The energy consumption of the transmitter and receiver node were measured based on the first order radio model. Equations (1) and (2) are used for measuring the energy required for transmitting and receiving of l bits packet at the distance of d.

(1) $$E_{TX}\left(l,d\right)=\{\begin{array}{c} l\times E_{elec}+l\times \varepsilon_{fs}\times d^{2}\;if\;d\leq d_{0}\\ l\times E_{elec}+l\times \varepsilon_{mp}\times d^{4}\;if\;d>d_{0} \end{array}$$

(2) $$E_{RX}\left(l,d\right)=l\times E_{elec}$$

where $E_{elec}$ specifies the energy utilized for transmission/reception, and $d_{0}$ specifies the threshold distance, which is expressed by Equation (3).

(3) $$d_{0}=\sqrt{\frac{\varepsilon_{fs}}{\varepsilon_{mp}}}$$

where the$\;\varepsilon_{mp}$ and$\;\varepsilon_{fs}$ are the amplification energy for multipath model and free space, respectively. The model of transmitter amplifier defines $\varepsilon_{fs}\;$and $\;\varepsilon_{mp}$.

### 4.3. Overview of Red Fox Optimization (RFO)

The red fox is an effective hunter of small creatures, including both wild and domestic animals. There are two types of red foxes: those who leave well-defined territories and those who live nomadic lives. Each herd shears a specific territory under the alpha couple’s system. If the chances of acquiring control of another territory are favorable when the young reach adulthood, they may elect to leave the herd and start their own herd. They stay in the family, or else ultimately receive their blood relation fox hunting land [27,28].

#### 4.3.1. Fundamental Principle

Each population is characterized by an n-coordinate point $\bar{x}=\left(x_{0},x_{1}\ldots \ldots x_{n-1}\right)$. In the symbolization ${\left({\bar{x}}_{j}^{i}\right)}^{t}\;$, i is fox count in the population and j is the coordinate to identify each fox ${\bar{x}}^{i}$ in iteration t. Let $f\in {\;R}^{n}$ be the standard character; ($\bar{x}{)}^{\left(i\right)}$ = $\left[{\left(x_{0}\right)}^{\left(i\right)},{\left(x_{1}\right)}^{\left(i\right)},\ldots {\left(x_{n-1}\right)}^{\left(i\right)}\right]\;$denotes the solution space dimensions. Each space is stated as ${\left(a,b\right)}^{n}$, a,b∈R. If the value of function $f(\left(\bar{x}{)}^{\left(i\right)}\right)$ is a global value on ($\bar{x}{)}^{\left(i\right)}$, this is the ideal solution (a,b).

#### 4.3.2. Global Exploration Stage

In a herd, each fox has a vital role to perform in the survival of the entire family. Members of the herd move to remote locations when there is no food in the local environment, or to explore new areas. As a result, the population is sorted first by fitness condition, and then the square of the Euclidean distance to each person in the population as ${\left({\bar{x}}^{best}\right)}^{t}$ is calculated using Equation (4).

(4) $$\mathrm{dis}\left({\left({\bar{x}}^{i}\right)}^{t},{\left({\bar{x}}^{best}\right)}^{t}\right)=\sqrt{{\left({\bar{x}}^{i}\right)}^{t}-{\left({\bar{x}}^{best}\right)}^{t}}$$

The transfer of individuals of the population in the optimal direction is represented as Equation (5).

(5) $${\left({\bar{x}}^{i}\right)}^{t}={\left({\bar{x}}^{i}\right)}^{t}+\alpha \mathrm{sign}\left({\left({\bar{x}}^{best}\right)}^{t}-{\left({\bar{x}}^{i}\right)}^{t}\right)$$

For all individuals in the population, $\alpha \in \;\mathrm{dis}\left({\left({\bar{x}}^{i}\right)}^{t},{\left({\bar{x}}^{best}\right)}^{t}\right)$ is a indiscriminately designated, ascending hyper-parameter, which is set after the iterative count.

#### 4.3.3. Traversing through the Local Habitat–Local Search Phase

The arbitrary value μ∈(0,1) was formerly set to mimic the chance of a fox being observed while moving closer to the prey, which defines the fox’s action as Equation (6)

(6) $$\{\begin{array}{c} \mathrm{Move}\;\mathrm{closer}\;\mathrm{if}\;\mu >0.75\\ \mathrm{Stay}\;\mathrm{and}\;\mathrm{disguise}\;\mathrm{if}\;\mu \leq 0.75 \end{array}$$

While utilizing an improved Cochleoid equation to depict the individual effort, μ displays constraint in transferring the population in this iteration [29]. For this drive, a∈(0, 0.2) signifies the scaling constraint chosen in repetition for all members of a population to arbitrarily represent varying locations, from the target through to fox arrival, and $\varphi_{0}\in \left(0,\;2\pi \right)$ is chosen for all animals at the start of the method to simulate the fox viewing angle. It helps determine the foraging fox’s eyesight radius, which is expressed as Equation (7).

(7) $$R=\left(\begin{array}{cc} a\frac{\sin\varphi_{0}}{\varphi_{0}} & if\;\varphi_{0}\neq 0\\ \theta & if\;\varphi_{0}\neq 0 \end{array}\right)$$

where θ is a random value between 0 and 1. Exemplary activities of the system spatial coordinates are mentioned in Equation (8):

(8) $$\{\begin{array}{c} x_{0}^{new}=ar\cdot \cos\left(\varphi_{1}\right)+x_{0}^{actual}\\ x_{1}^{new}=ar\cdot \sin\left(\varphi_{1}\right)+ar\cdot \cos\left(\varphi_{2}\right)+x_{1}^{actual}\\ x_{2}^{new}=ar\cdot \sin\left(\varphi_{1}\right)+ar\cdot \sin\left(\varphi_{2}\right)+ar\cdot \cos\left(\varphi_{3}\right)+x_{2}^{actual}\\ \ldots \ldots \\ x_{n-2}^{new}=ar\cdot \sum_{k=1}^{n-2}\sin\left(\varphi_{k}\right)+ar\cdot \cos\left(\varphi_{n-1}\right)+x_{n-2}^{actual}\\ x_{n-1}^{new}=ar\cdot \sin\left(\varphi_{1}\right)+ar\cdot \sin\left(\varphi_{2}\right)+\ldots \;+ar\cdot \sin\left(\varphi_{n-1}\right)+x_{n-1}^{actual} \end{array}$$

Every angular assessment is randomized, conferring to $\varphi_{1},\varphi_{1},\ldots .\varphi_{n-1}\;\in \left(0,2\pi \right)$.

#### 4.3.4. Reproduction Stage

In order to keep the population size constant, the two strongest characters ${\left({\bar{x}}^{\left(1\right)}\right)}^{t}\;\mathrm{and}\;\;{\left({\bar{x}}^{\left(2\right)}\right)}^{t}$ are chosen, to signify the alpha link, Equation (9) calculates the habitat center:

(9) $${\mathrm{habitat}}^{{\left(\mathrm{center}\right)}^{t}}=\frac{{\left({\bar{x}}^{\left(1\right)}\right)}^{t}+{\left({\bar{x}}^{\left(2\right)}\right)}^{t}}{2}$$

The square habitat of the distance amongst the defined parameters is expressed as Equation (10):

(10) $${\mathrm{habitat}}^{{\left(\mathrm{diameter}\right)}^{t}}=\sqrt{\left\|{\left({\bar{x}}^{\left(1\right)}\right)}^{t}-{\left({\bar{x}}^{\left(2\right)}\right)}^{t}\right\|}$$

Equation (10), shown above, shows the distances between the defined parameters. All classifications of points are reverted through the optimization process, and limited by random values, which is the optimal solution in a specified iteration. In every iteration, function is expressed in the sense of distance among the alpha function. For every iterative count, a random constraint is considered as k∈(0, 1), which expresses substitutions with respect to Equation (11):

(11) $$\{\begin{array}{c} \mathrm{New}\;\mathrm{nomadic}\;\mathrm{individual}\;\mathrm{if}\;k\geq 0.45\\ \mathrm{Reproductiom}\;\mathrm{of}\;\mathrm{the}\;\mathrm{alpha}\;\mathrm{couple}\;\mathrm{if}<0.45 \end{array}$$

If reproducing an optimal solution for two individuals, ${\left({\bar{x}}^{\left(1\right)}\right)}^{t}$ and ${\left({\bar{x}}^{\left(2\right)}\right)}^{t}$ join in a fresh individual ${\left({\bar{x}}^{reproduced}\right)}^{t}$, as shown in Equation (12):

(12) $${\left({\bar{x}}^{reproduced}\right)}^{t}=k\frac{{\left({\bar{x}}^{\left(1\right)}\right)}^{t}+{\left({\bar{x}}^{\left(2\right)}\right)}^{t}}{2}$$

The time complexity of RFO is analyzed here. The population size is n, problem dimension is D, and the number of iterations is T. In every iteration, all entities are organized, and produce an $\;O\;{\left(n\times D\right)}^{2}$ function. The time complexity of RFO is minimized through a quick sorting process, particularly for high dimensional tasks. Neglecting the terms that exist in low-order, the computational time is demarcated as $O\left(3\times T\times n^{2}\times D^{2}\right)\;$functions.

## 5. Proposed Method

**Figure 2:**
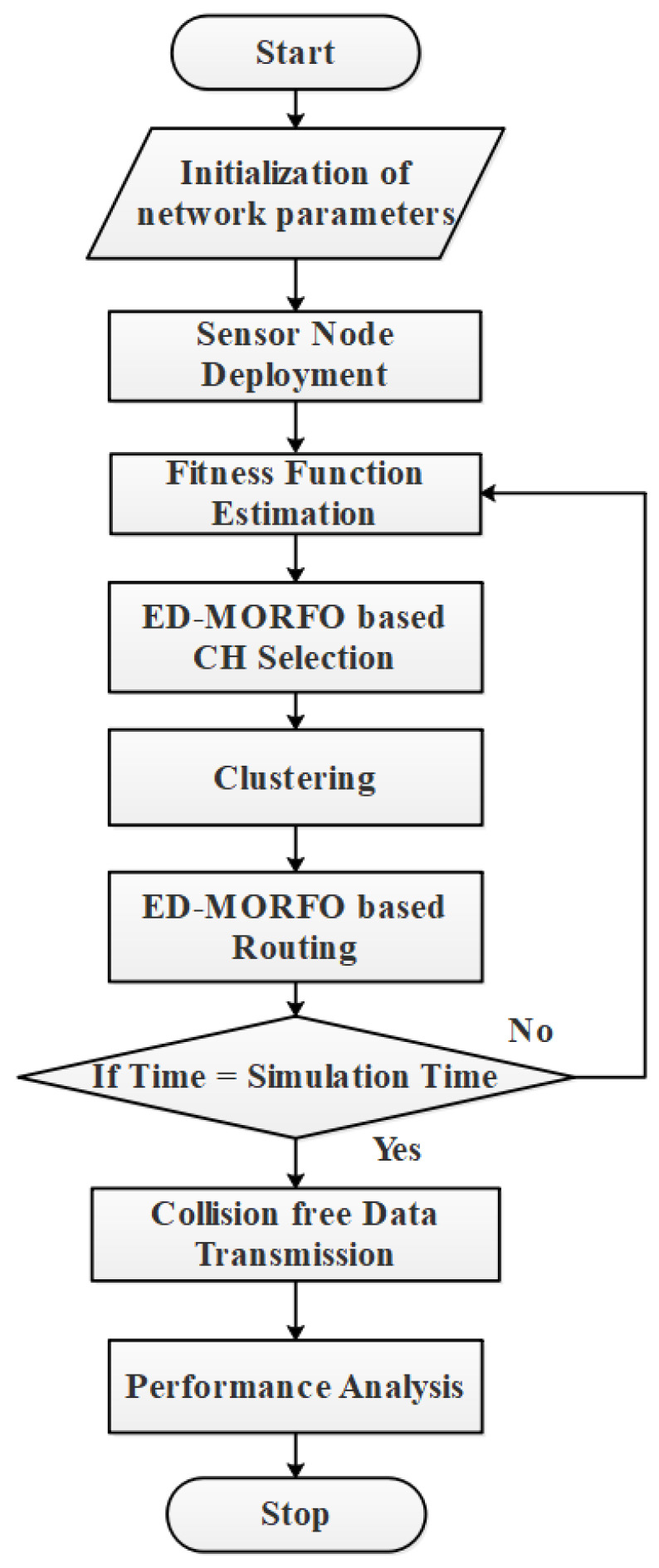
Flowchart of clustering and routing using ED-MORFO.

ED-MORFO was used to establish clustering and routing in this study. The algorithm’s searching capabilities were combined with the fitness function values. Four different fitness function parameters (residual energy, distance, network coverage, and the degree of nodes) were taken into account throughout the clustering process [30]. By considering two alternative distance functions, the network’s energy usage was considerably reduced. Furthermore, node failure was avoided in the transmission path, by taking into account the nodes’ remaining energy. By preventing node failure, packet loss when data are transmitted was minimized. The major goal of this study was to reduce energy depletion, in order to extend the network’s lifespan. Figure 2 depicts a general flowchart of clustering and routing.

The steps for the flowchart are as follows:

- A clustering technique is designed to distribute the system into groups;
- The nodes are arbitrarily placed in the concerned zone at first, then mobile nodes are indicated as a dynamic that is entirely reliant on the position of the node. ED-MORFO is used to cluster networks in this case. At that moment, the CH is determined, based on the distance between neighbors, residual energy, and the distance to the base station location, among other factors;
- Routing methods generated using the suggested ED-MORFO are used to create the ideal path between the CH and BS;
- Starting with the routing process, an ideal node is identified to provide the definite route from the CH to BS;
- Once the path from the source to the destination is established, the source node sends the data in the destination route. This ED-MORFO calculates the best route by taking into account numerous objective functions such as residual energy, the distance between the CH and BS, and hop count;
- BS is frequently used to observe the residual energy of nodes. To avoid network packet loss, re-clustering/rerouting is performed frequently.

### 5.1. ED-MORFO-Based CH Selection

In this research, an efficient technique called ED-MORFO was developed, for the clustering and routing process. In the cluster and routing procedure, non-CH nodes join a CH, using fitness function parameters. In a conventional process, non-CH nodes simply attach to the CH by deliberating distance factor, which could produce an imbalanced load of the CHs, and lead to higher energy consumption of the network. Here, the proposed ED-MORFO is employed for the clustering and routing process with various fitness function parameters to produce a lower computational complexity, high stability, speed of optimization, and low time complexity.

The fundamental purpose of an ED-MORFO-based clustering procedure is to select the best number of nodes in the neighborhood, such as CHs. The goal is to achieve proper fitness by calculating residual energy, distance, network coverage, and node degree.

#### Fitness Function Derivation

- Residual energy (RE)

RE [31] is characterized in Equation (13):


(13) $$RE=\sum_{i=1}^{m}\frac{1}{E_{CHi}}$$

b. Inter and intra cluster distance (*D*)

This section explains the distance between each CH and BS. As previously stated, while considering energy usage, the sensor node is fully controlled by the transmission distance. When the base station is further away from the mobile node, it requires more energy to complete the procedure [31]. As a result, in the network, the cluster head with the shortest Euclidean distance starting from the BS is most favored. So, the inter cluster and intra cluster objectives are mentioned as D1 and D2, which can be minimized and expressed in Equations (14) and (15).

(14) $$D1=\sum_{i=1}^{m}\left(dis1\;\left({\mathrm{CH}}_{j},\;\mathrm{BS}\right)\right)$$

(15) $$D2=\sum_{j=1}^{q}\left(\sum_{i=1}^{cm_{j}}dis2\;\left(s_{i},{\mathrm{CH}}_{j}\right)/cm_{j}\right)\;$$

where the $cm_{j}$ represents the amount of nodes present in the cluster and $dis2\left(s_{i},{\mathrm{CH}}_{j}\right)$ represents the distance between the sensor i and jth CH.

- Network coverage

The network coverage [31] is defined by this Equation (16):

(16) $$N_{cov}=r\left(N_{i}\right)$$

where $r\left(N_{i}\right)\;$represents the radius covered by node. The objective is represented as

$$N_{cov}=\frac{1}{N_{T}}\sum_{i=1}^{N}N_{cov}\left(N_{i}\right)$$

b. Degree of nodes

It is demarcated as the amount of non-CH applicants [31] who go to the specific portable node. $D_{N}$ is expressed in Equation (17).

(17) $$D_{N}=\sum_{i=1}^{m}I_{i}$$

Accordingly, the normalization process (F(x)) is exploited to every objective $\alpha_{1},\alpha_{2},$$\alpha_{3},\alpha_{4}$,$\;\alpha_{5}$, which is shown in Equation (18).

(18) $$F\left(x\right)=\frac{f_{i}-f_{min}}{f_{max}-f_{min}}$$

where function value is signified as $f_{i}$, and $f_{i}$ and $\;f_{max}$ are specified as the minimum and maximum fitness values, respectively. Fitness function is established in a way that means a trade-off is maintained inside the specified objectives. Finally, the distinct objectives are converted as a single objective function through the addition of multiplied values. A multi-objective fitness function is now established by means of ED-MORFO, which is given in Equations (19) and (20).


(19) $$fitness=\alpha_{1}RE+\alpha_{2}D1+\alpha_{3}D2+\alpha_{4}N_{cov}+\alpha_{5}D_{N}$$

(20) $$\mathrm{where}\;\sum_{i=1}^{5}\alpha_{i}=1;\mathrm{and}\;\alpha_{i}\in \left(0,1\right)$$

$\alpha_{i}$ is stated as weighted parameter, and each dimension of a weighted parameter is initialized through a random number amongst 0 and 1, which is assigned to each fitness function [31] as ($\alpha_{1}=0.4,\;\alpha_{2}=0.3,\;\alpha_{3}=0.2,\;\alpha_{4}=0.05,\alpha_{5}=0.05$). The transmission distance across the WSN is minimized by taking into account both the distance and the residual energy when choosing the node with the highest remaining energy. As a result, these fitness functions are used to determine the best data transmission route. After selecting the CH, the clusters form according to distance and energy. While in the process of routing, existing routing algorithms have fitness functions and minimization processes based on the distances to the CH only. However, in this proposed ED-MORFO, along with the distances, additional fitness function parameters such as queue length and link quality are included, to minimize the energy consumption and extend the network lifetime. When the routing is recognized by means of ED-MORFO, the transmission of the data packet is more efficient. To generate/transmit data over the network, the queue length utilized in routing is employed. The link quality is then utilized to determine the route’s value, based on the transmitted and received packets.

### 5.2. Routing Using ED-MORFO

The primary goal of this study was to find a neighboring optimum path from each cluster head to the appropriate BS. Source CH collects the message from the nearby nodes once the routing path is created. The data transmission is initiated through the network after the routing path is generated. The fitness function procedures are described in detail.

#### 5.2.1. Initialization

Each ED-MORFO in routing represents the data sending route between every CH and BS. The proposed transmission path between the source and BS modifies every time in the routing process. The quantity of CHs in the associated transmission route is equal to the measurement of each fox.

#### 5.2.2. Fitness Function

The parameters used in the routing optimization are mentioned as follows:

- Queue length

It deliberates the congestion limit of every node present in WSN. The QL indicated in Equation (21) is regarded as a primary fitness value during routing. This QL is utilized to increase data delivery performance, because the created ED-MORFO requires sending alert messages over the WSN.

(21) $$\mathrm{QL}=\frac{RP_{k}}{Total\;buffer}$$

where the received packets at k-th node is represented as $RP_{k}$.

b. Link quality

Link quality is exploited to determine effective data transmission among the nodes l and k. The Equation (22) is utilized to determine link quality.

(22) $$\mathrm{Link}\;\mathrm{quality}=\frac{1}{f\times r}\;$$

where f and r define the forward and reverse data transmission among the nodes, respectively.

The details about the distance (D1 and D2) and residual energy (RE) are already defined in the section. Consequently, all the different objective fitness values are in conflict, and they are transformed into a single objective function, which is illustrated in Equation (23):

(23) $$\begin{array}{c} Routing\;fitness=\delta_{1}\times \mathrm{QL}+\delta_{2}\times \mathrm{Link}\;\mathrm{quality}+\delta_{3}\times D1+\delta_{4}\times D2\\ \;+\delta_{5}\times RE \end{array}$$

where $\delta_{1},\;\delta_{2},\;\delta_{3},\delta_{4}$, and $\delta_{5}$ are the weighted parameters associated with each objective, which is equal to 0.3, 0.2, 0.15, 0.15, and 0.2, respectively [31].

## 6. Results and Discussion

This segment clearly describes the outcomes of proposed ED-MORFO approach. MATLAB simulation is used to implement the ED-MORFO, which runs on a 4 GB RAM machine, with an Intel Core PC. The goal of this investigation is to wisely control the energy depletion suggested via the routing method. Four performances, specifically distance, overhead, latency, and packet size, contribute to the proposed calculation to conduct advancement. Table 1 shows the simulation specification for the proposed system.

### 6.1. Performance of Energy Consumption

**Figure 3:**
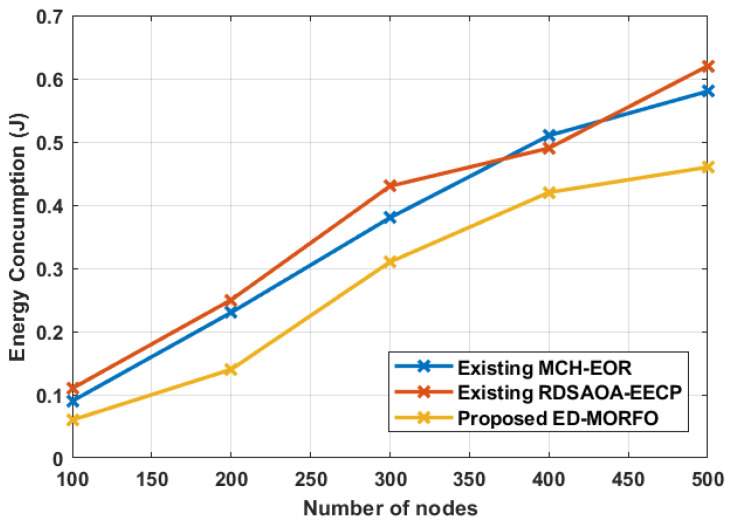
Performance of energy consumption for proposed EDMORFO and Existing MCH-EOR [24], RDSAOA-EECP [25].

The comparison of the suggested ED-MORFO and correlated devices, such as the existing MCH-EOR [24] and existing RDSAOA-EECP [25], in the case of energy consumption, is shown in Figure 3. Figure 3 shows that when compared to the following conventional approaches, the suggested ED-MORFO consumes less energy. In the ED-MORFO technique, a smaller number of nodes are twisted in packet progression. Furthermore, more energy is saved, as nodes with the highest optimality factor continue to be important in progressing data packets; in contrast, the traditional methods require an extra node when dispatching the same data packet; as a result, sophisticated energy is consumed in the existing MCH-EOR [24] and existing RDSAOA-EECP [25] methods. The analysis of energy consumption performance is shown in Table 2. It shows that the performance of the proposed ED-MORFO varies from 0.06 to 0.46, while the existing MCH-EOR [24] achieves 0.09 to 0.58, and RDSAOA-EECP [25] achieves 0.11 to 0.62.

### 6.2. Performance of PDR

**Figure 4:**
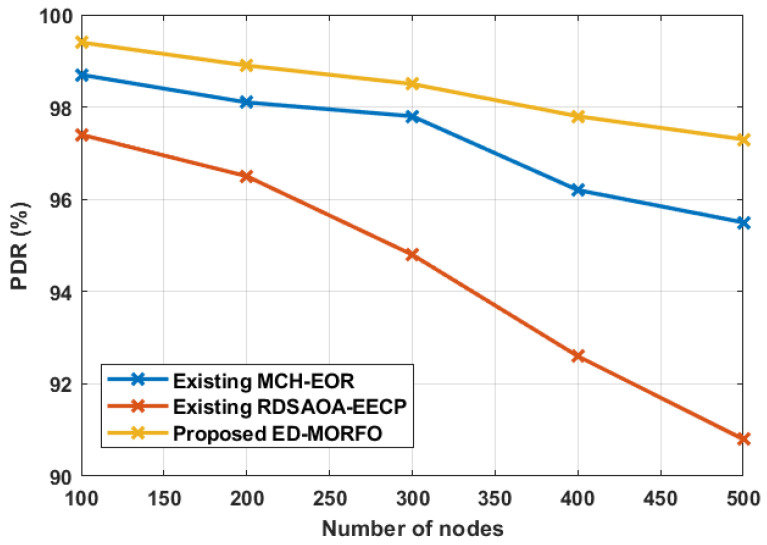
Performance of PDR for proposed EDMORFO and Existing MCH-EOR [24], RDSAOA-EECP [25].

Figure 4 shows the results of PDR for both planned and existing technologies. When the number of nodes increases, the size of the routing path results in an increase in delay. The performance comparison for the PDR is shown in Table 3. Table 3 clearly illustrates that the suggested ED-MORFO achieves a higher PDR of 99.4%, whereas the PDR of the existing MCH-EOR [24] attains 98.7%, and RDSAOA-EECP [25] achieves a PDR of 97.4%.

### 6.3. Performance of Throughput

**Figure 5:**
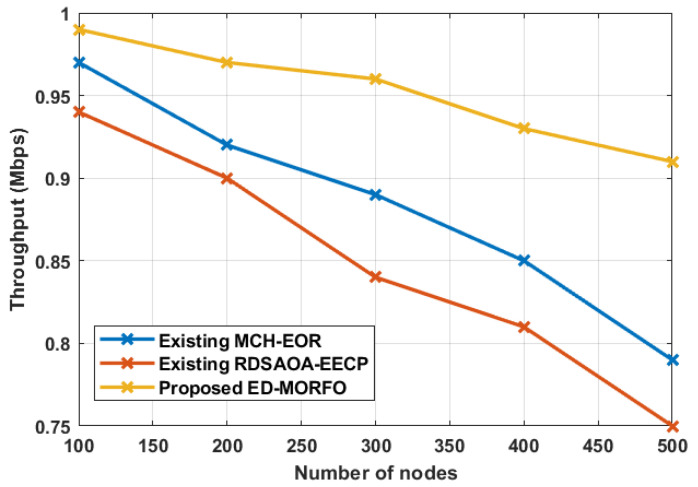
Performance analysis of throughput for proposed EDMORFO and Existing MCH-EOR [24], RDSAOA-EECP [25].

The outcomes of the throughout achievement for both recommended and present techniques are shown in Figure 5. The suggested ED-MORFO obtains superior outcomes in terms of throughput, compared to the existing MCH-EOR [24] and existing RDSAOA-EECP [25]. As ED-MORFO has a long network lifespan, the base station receives more data packets. Table 4 shows the performance analysis for throughput. Table 4 shows that the proposed ED-MORFO achieves a maximum throughput of 0.99 Mbps, whereas the existing MCH-EOR [24] only manages 0.97 Mbps, and RDSAOA-EECP [25] only manages 0.94 Mbps.

When compared to the existing approaches, the total simulation results show that the suggested ED-MORFO achieves better results in all node counts (100–500).

### 6.4. Performance of Network Lifetime

**Figure 6:**
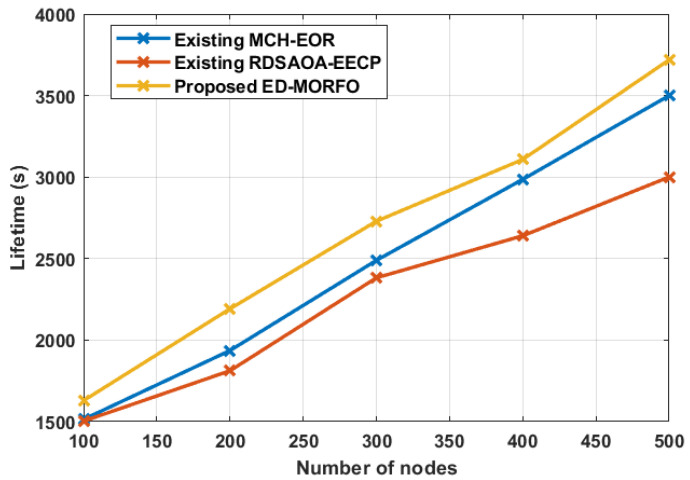
Performance of network lifetime for proposed EDMORFO and Existing MCH-EOR [24], RDSAOA-EECP [25].

ED-MORFO and existing methodologies were used to compare the performance of the lifetime of a network. Figure 6 depicts the existing MCH-EOR [24] and RDSAOA-EECP [25] assessments. Figure 6 illustrates that, when compared to existing approaches, ED-MORFO produces better outcomes. In contrast to the existing MCH-EOR [24] and RDSAOA-EECP [25], there is an increase in the longevity of ED-MORFO. As the network’s node count grows, additional sensor nodes begin steering packets indiscriminately, and there is a significant chance that nodes perish at some point. Only the optimal node is assigned to transfer packets in the ED-MORFO approach, resulting in increased battery life and network longevity. Table 5 summarizes the results of the lifetime performance analysis. When compared to existing methodologies, the suggested ED-MORFO improves the network lifetime at a specific node, as shown in the table. The suggested ED-MORFO achieves a network lifetime of 3719 s, compared to 3500 s for the MCH-EOR [24], and 3000 s for the RDSAOA-EECP [25].

### 6.5. Performance of End-to-End Delay

**Figure 7:**
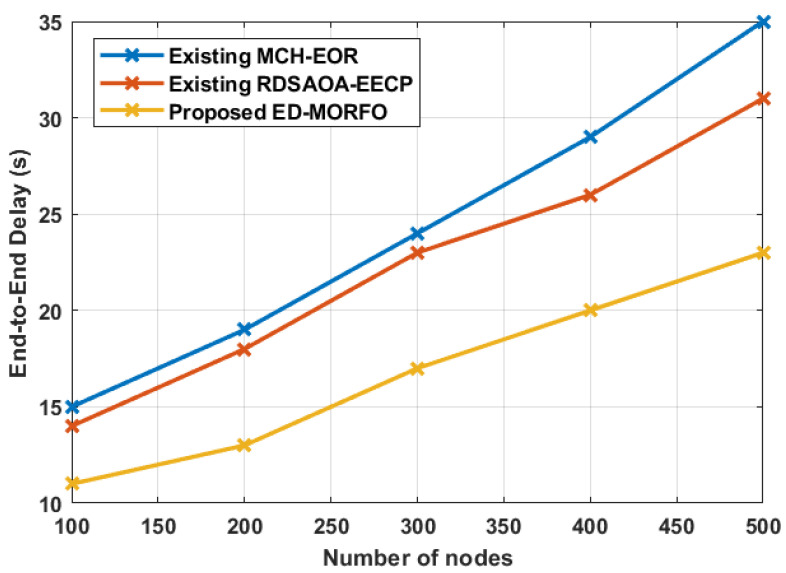
Performance of end-to-end delay for proposed EDMORFO and Existing MCH-EOR [24], RDSAOA-EECP [25].

ED-MORFO and existing methodologies were used to compare the performance of the lifetime of a network. Figure 7 depicts the existing MCH-EOR [24] and RDSAOA-EECP [25] assessments. Figure 7 illustrates that, when compared to existing approaches, ED-MORFO produces better outcomes. In contrast to the existing MCH-EOR [24] and RDSAOA-EECP [25], there is a decrease in the end-to-end delay of ED-MORFO. Figure 7 clearly illustrates that ED-MORFO achieves the least delay, and continues to try to transmit the data packet to the best intermediary node, satisfying the optimality feature. On the contrary, the traditional MCH-EOR [24] and RDSAOA-EECP [25] methods take a long time to determine the end point node. Furthermore, it initiates the prolonged return after the end point node. As a result, the related system’s typical end-to-end delay is longer than the ED-MORFO approach. Table 6 tabulates the comparative analysis of end-to-end delay performance. Table 6 shows that the proposed ED-MORFO achieves a time delay of 11 s when compared with the existing techniques of MCH-EOR and RDSAOA-EECP, which attain 15 s and 14 s, respectively.

### 6.6. Performance of Routing Overhead

**Figure 8:**
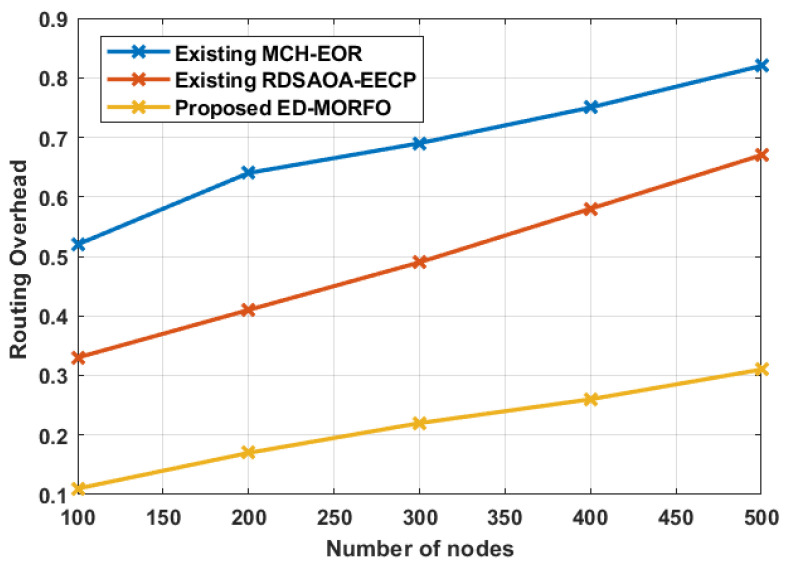
Performance of routing overhead for proposed EDMORFO and Existing MCH-EOR [24], RDSAOA-EECP [25].

ED-MORFO and existing methodologies were used to compare the performance of routing overhead. Figure 8 depicts the existing MCH-EOR [24] and RDSAOA-EECP [25] assessments. Figure 8 illustrates that, when compared to existing approaches, ED-MORFO produces better outcomes. In contrast to the existing MCH-EOR [24] and RDSAOA-EECP [25], there is a decrease in the routing overhead of ED-MORFO. Table 7 tabulates the comparative analysis of routing overhead performance. Table 7 shows that the proposed ED-MORFO achieves a lower routing overhead of 0.11 at 100 nodes, which is better when compared with the existing techniques of MCH-EOR and RDSAOA-EECP, which attain 0.52 and 0.33, respectively.

### 6.7. Performance of Packet Loss Rate

**Figure 9:**
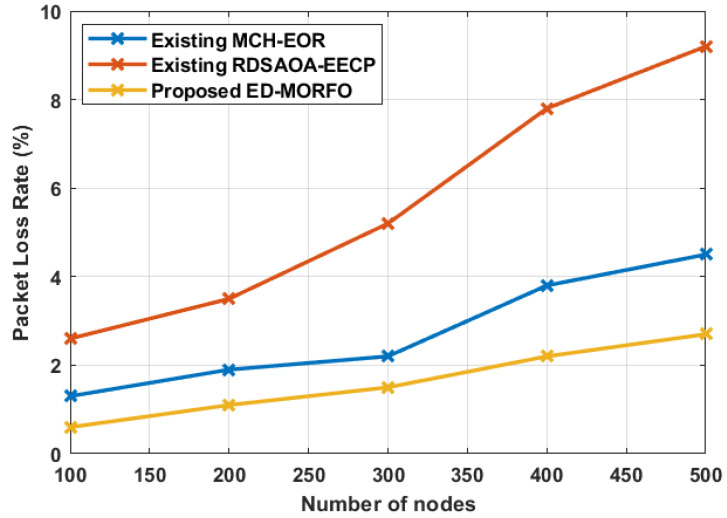
Performance of PLR for proposed EDMORFO and Existing MCH-EOR [24], RDSAOA-EECP [25].

ED-MORFO and existing methodologies were used to compare the performance of packet loss rate (PLR). Figure 9 depicts the existing MCH-EOR [24] and RDSAOA-EECP [25] assessments. Figure 9 illustrates that, when compared to existing approaches, ED-MORFO produces better outcomes. In contrast to the existing MCH-EOR [24] and RDSAOA-EECP [25], there is a decrease in the packet loss rate of ED-MORFO. Table 8 tabulates the comparative analysis of packet loss rate performance. Table 8 shows that the proposed ED-MORFO achieves a better PLR of 0.6% when compared with the existing techniques of MCH-EOR and RDSAOA-EECP, which attain rates of 1.3% and 2.6%, respectively.

### 6.8. Performance of Computational Complexity

**Figure 10:**
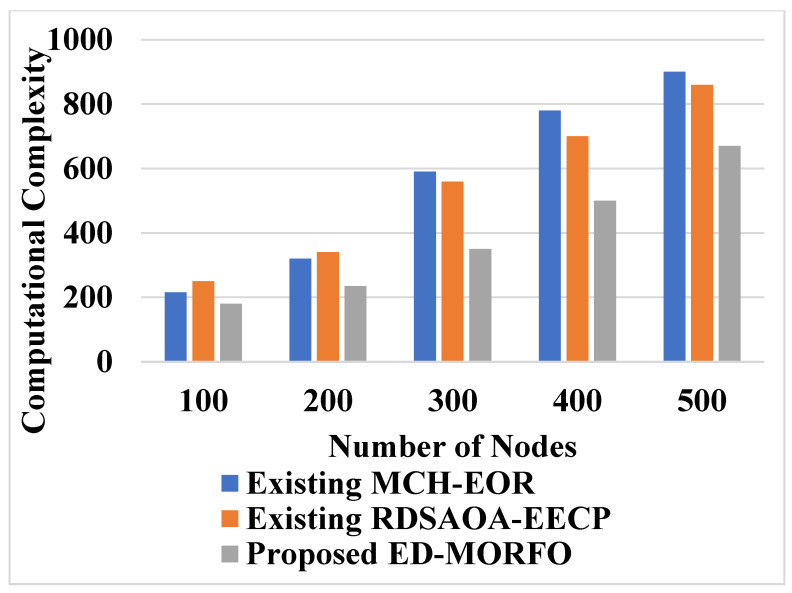
Performance of computational complexity for proposed EDMORFO and Existing MCH-EOR (Mehta, and Saxena, 2020), RDSAOA-EECP (Rajeswarappa, and Vasundra, 2021).

The computational complexity analysis for the existing MCH-EOR [24], RDSAOA-EECP [25], and the proposed ED-MORFO are calculated here. The complexity range is high when the number of nodes is high. Table 9 shows the performance of computational complexity. Table 9 clearly shows that the proposed ED-MORFO has less computational time when compared to the existing MCH-EOR [24] and RDSAOA-EECP [25]. Figure 10 shows the graphical analysis of computational complexity.

Based on the results of the comparison, it is demonstrated that the ED-MORFO technique outperforms the existing techniques of MCH-EOR [24] and RDSAOA-EECP [25]. Due to an incorrect fitness function consideration during CH selection, existing CDASs achieve a worse performance. In ED-MORFO clustering, different fitness variables are used to discover an appropriate CH between the sensors. Following that, an appropriate route creation, using the ED-MORFO, is applied to reduce the node’s energy depletion. As a result, when compared to existing methods, the proposed ED-MORFO gain a longer network lifetime. The increased lifetime of the ED-MORFO is due to the greater volume of data packets sent to the BS. Similarly, the mean time complexity of the existing RDSAOA-EECP [25] is lower in the computational burden of 0.1912 s, but the energy consumptions protocol with different densities of sensor nodes that require improvement is minimized. The proposed ED-MORFO shows a lower computational burden of 0.10 s, as the algorithm locates the individuals in the direct surroundings of the optimum, once the short interval of time is provided.

## 7. Conclusions

In this research, the ED-MORFO algorithm is proposed, analyzed, and well-organized to recognize the energy-efficient clustering and routing in a WSN. Here, the ED-MORFO is recommended to reach the base station with minimum energy consumption. Initially, the CH selection is based on a variety of criteria; the primary goal of improving the network lifetime is achieved while lowering energy consumption. After choosing the CH, the proposed EDMORFO algorithm is used to choose the best route for transmitting data to the BS; thus, the energy-efficiency of the WSN is improved. The proposed ED-MORFO combination is tested for a system size, with node counts ranging from 100 to 500, and the energy efficiency is investigated through network analysis using PDR, energy consumption, throughput, and network lifetime. In contrast to traditional and cluster-dependent routing systems, such as MCH-EOR and RDSAOA-EECP, the simulated outcomes of ED-MORFO are superior. According to the simulation results, the proposed ED-MORFO outperforms existing protocol systems in all characteristics, by reducing energy consumption by up to 0.46 J. It also achieves a PDR of 99.4%, a PLR of 0.6%, an end-to-end delay of 11 s, a routing overhead of 0.11, a maximum throughput of 0.99 Mbps, and a network lifetime of up to 3719 s. In the future, the proposed approach can be tested with various specification parameters and a variable number of node counts; in addition, a hybrid optimization-based routing procedure can be implemented to achieve better energy consumption results.

## Figures and Tables

**Table 1:** Simulation specification.

Parameter	Value
Transmission power	0.660 W
Receiving power	0.4 W
Packet size	512 bytes
Number of nodes	200
Initial energy	14.0 J
Antenna model	Omni antenna
Data transfer rate	250 (Kb/s)
Network size	1000 × 1000 m
Simulation time	100 s
Traffic type	CBR
Transmission rate	50 to 250 Kb/s

**Table 2:** Performance of energy consumption.

Number of Nodes	Energy Consumption (J)		
	Existing MCH-EOR [24]	Existing RDSAOA-EECP [25]	Proposed ED-MORFO
100	0.09	0.11	0.06
200	0.23	0.25	0.14
300	0.38	0.43	0.31
400	0.51	0.49	0.42
500	0.58	0.62	0.46

**Table 3:** Performances of PDR.

Number of Nodes	PDR (%)		
	Existing MCH-EOR [24]	Existing RDSAOA-EECP [25]	Proposed ED-MORFO
100	98.7	97.4	99.4
200	98.1	96.5	98.9
300	97.8	94.8	98.5
400	96.2	92.6	97.8
500	95.5	90.8	97.3

**Table 4:** Performances of throughput.

Number of Nodes	Throughput (Mbps)		
	Existing MCH-EOR [24]	Existing RDSAOA-EECP [25]	Proposed ED-MORFO
100	0.97	0.94	0.99
200	0.92	0.90	0.97
300	0.89	0.84	0.96
400	0.85	0.81	0.93
500	0.79	0.75	0.91

**Table 5:** Performances of network lifetime.

Number of Nodes	Network Lifetime (s)		
	Existing MCH-EOR [24]	Existing RDSAOA-EECP [25]	Proposed ED-MORFO
100	1514	1503	1629
200	1936	1812	2192
300	2489	2382	2729
400	2986	2640	3108
500	3500	3000	3719

**Table 6:** Performances of end-to-end delay.

Number of Nodes	End-to-End Delay (s)		
	Existing MCH-EOR [24]	Existing RDSAOA-EECP [25]	Proposed ED-MORFO
100	15	14	11
200	19	18	13
300	24	23	17
400	29	26	20
500	35	31	23

**Table 7:** Performances of routing overhead.

Number of Nodes	Routing Overhead		
	Existing MCH-EOR [24]	Existing RDSAOA-EECP [25]	Proposed ED-MORFO
100	0.52	0.33	0.11
200	0.64	0.41	0.17
300	0.69	0.49	0.22
400	0.75	0.58	0.26
500	0.82	0.67	0.31

**Table 8:** Performances of packet loss rate.

Number of Nodes	Packet Loss Rate (%)		
	Existing MCH-EOR [24]	Existing RDSAOA-EECP [25]	Proposed ED-MORFO
100	1.3	2.6	0.6
200	1.9	3.5	1.1
300	2.2	5.2	1.5
400	3.8	7.8	2.2
500	4.5	9.2	2.7

**Table 9:** Performances of computational complexity.

Number of Nodes	Computational Complexity		
	Existing MCH-EOR [24]	Existing RDSAOA-EECP [25]	Proposed ED-MORFO
100	215	250	180
200	320	340	235
300	590	559	350
400	780	700	500
500	900	860	670

## Data Availability

No new data were created or analyzed in this study. Data sharing is not applicable to this article.
